# Takotsubo Cardiomyopathy in a Patient Undergoing 5-Fluorouracil Infusion: Considerations for Folinic Acid, 5-Fluorouracil, and Oxaliplatin (FOLFOX) Cardiotoxicity and Rechallenge

**DOI:** 10.7759/cureus.82145

**Published:** 2025-04-12

**Authors:** Nabeel Sami, Amit Raizada, Gabriella Jade Phillip, Dhara Rana, Shudhanshu Alishetti

**Affiliations:** 1 Internal Medicine, New York-Presbyterian Brooklyn Methodist Hospital, New York, USA; 2 Internal Medicine, Inspira Health Network, Vineland, USA; 3 Cardiology, New York-Presbyterian Brooklyn Methodist Hospital, New York, USA

**Keywords:** 5-fluorouracil, cardiotoxicity, colorectal cancer, coronary artery vasospasm, folfox-6, takotsubo cardiomyopathy

## Abstract

Takotsubo cardiomyopathy (TCM) is a rare but reversible cause of transient ventricular dysfunction, often triggered by sympathetic stimulation from physical or emotional stress. Patients with a history of TCM undergoing cardiotoxic chemotherapy such as FOLFOX (folinic acid, 5-fluorouracil (5-FU), and oxaliplatin) require vigilant monitoring due to potential cardiac complications, including coronary vasospasm and direct myocardial toxicity. We present a case of progressively worsening TCM diagnosed via coronary angiography and left ventriculography three days after a patient received their first FOLFOX infusion for sigmoid adenocarcinoma. The cardiomyopathy resolved with conservative management, and one month later, the patient was admitted for a monitored FOLFOX rechallenge in the telemetry unit, which she tolerated well. This case highlights the importance of cardiac surveillance in patients with prior TCM requiring chemotherapy and discusses strategies for minimizing cardiotoxic risk during 5-FU rechallenge.

## Introduction

Takotsubo cardiomyopathy (TCM), also known as stress-induced cardiomyopathy or “broken heart syndrome,” is characterized by acute left ventricular (LV) dysfunction, often with apical ballooning and no evidence of obstructive coronary artery disease [1]. Patients typically present with moderately elevated cardiac biomarkers (troponin, creatine kinase) and markedly elevated brain natriuretic peptide (BNP) [1]. The underlying pathophysiology involves catecholamine-induced myocardial stunning, microvascular dysfunction, and increased sympathetic stimulation [1].

Certain chemotherapy regimens, including FOLFOX (folinic acid, 5-fluorouracil (5-FU), and oxaliplatin), have been associated with cardiotoxicity, primarily due to 5-FU, a backbone agent in colorectal cancer therapy. 5-FU is known to cause coronary vasospasm, myocarditis, and direct myocardial injury, though its precise mechanisms remain unclear [2]. While the relationship between FOLFOX and TCM is not firmly established, a possible association has been suggested in case reports. There have been instances of angina and rapid cardiac function recovery following 5-FU cessation, raising concerns about a potential link between 5-FU cardiotoxicity and TCM [3].

Rechallenging patients with 5-FU poses a significant risk of recurrent cardiotoxicity. One case series reported a 90% recurrence rate of angina or electrocardiogram (ECG) changes following rechallenge [4]. Additionally, a review of 377 cases found that 13% of rechallenged patients died from 5-FU-induced cardiotoxicity, compared to an 8% mortality rate after initial exposure [2]. It was unclear whether these deaths were due solely to cardiac events or other treatment-associated complications. These findings support the case for implementing risk reduction strategies in patients receiving 5-FU-based chemotherapy.

## Case presentation

**Figure 1:**
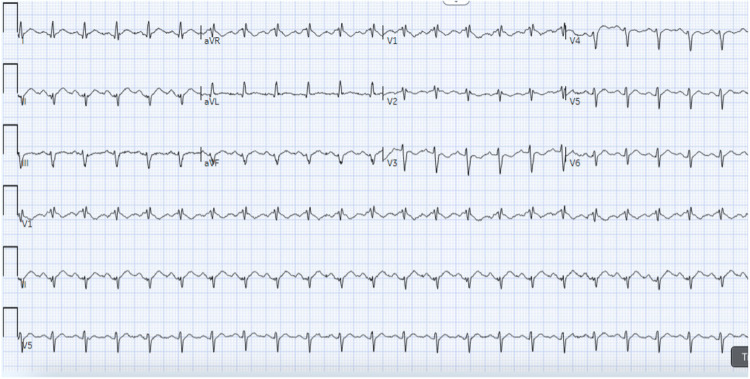
ECG taken on admission showing sinus tachycardia with nonspecific ST-segment and T-wave changes ECG: electrocardiogram

**Figure 2:**
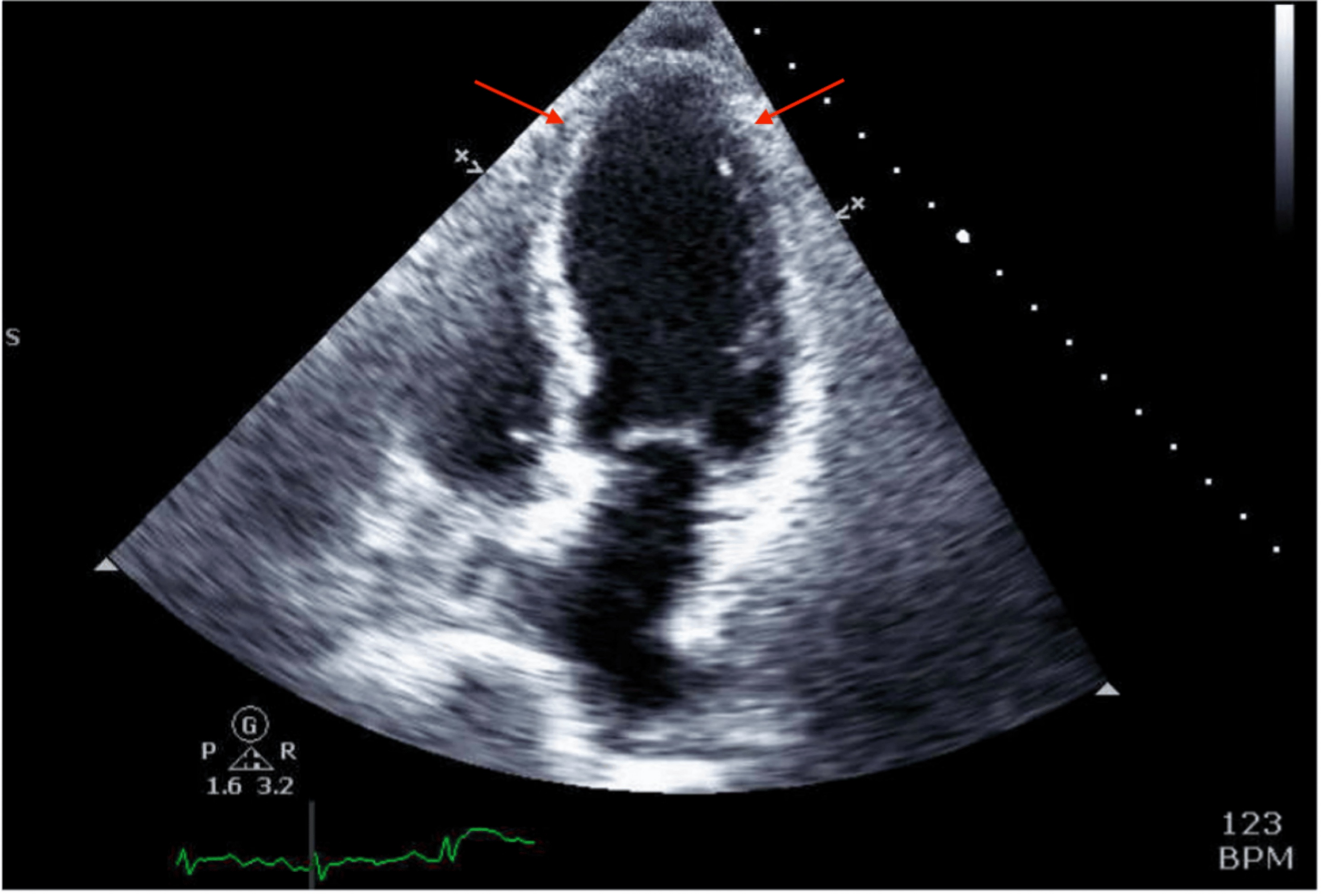
TTE in apical four-chamber view demonstrating LV apical hypokinesis Red arrows indicate the apical region of the left ventricle, which exhibits reduced wall motion consistent with apical hypokinesis. TTE: transthoracic echocardiogram, LV: left ventricular, BPM: beats per minute

A 74-year-old female with a history of severe anxiety and sigmoid adenocarcinoma who recently initiated FOLFOX chemotherapy presented with both chest pain and tachycardia three days after chemotherapy initiation. Her chest pain began one day after initiation and progressively worsened for two days. ECG demonstrated tachycardia without dynamic ischemic ECG changes with nonspecific ST-segment and T-wave changes (Figure 1). Laboratory workup showed an elevated troponin of 221 ng/L and a BNP of 1062 pg/mL. Transthoracic echocardiogram (TTE) demonstrated severe LV hypokinesis with a markedly reduced ejection fraction (LV ejection fraction (LVEF) <20%) (Figure 2).

**Figure 3:**
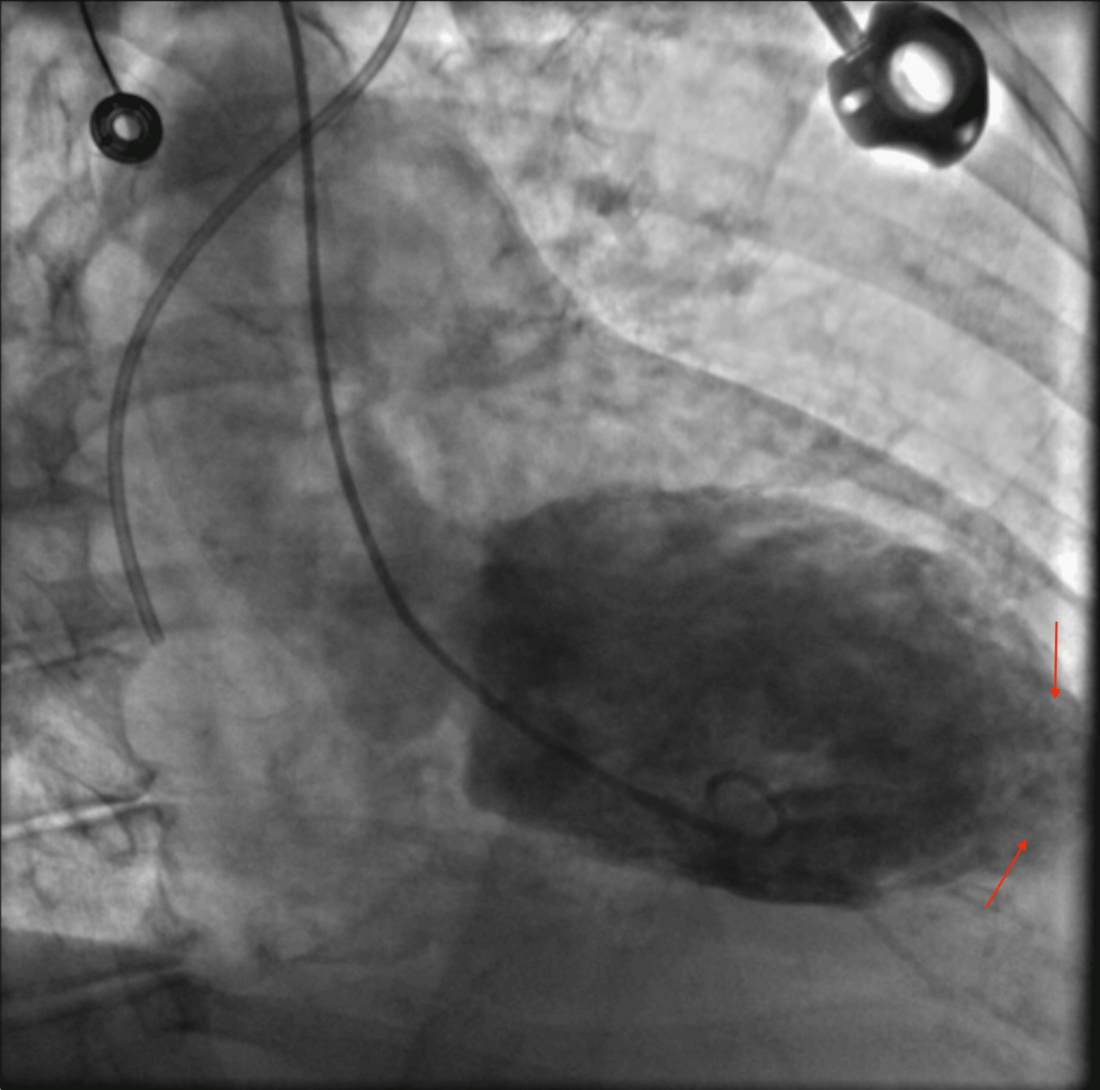
Left ventriculography confirming a severely reduced mid and apical LV systolic function with hyperkinetic basal segments and apical ballooning Contrast injection reveals akinesis of the mid and apical LV segments (red arrows), with compensatory hypercontractility of the basal myocardium. LV: left ventricular

Given her new-onset heart failure with reduced LVEF, she underwent coronary angiography, which did not demonstrate any significant obstructive coronary artery disease. Left ventriculography confirmed severely reduced mid to apical LV systolic function with hyperkinetic basal segments and apical ballooning, consistent with TCM (Figure 3). She was treated conservatively with medical therapy, including metoprolol succinate and losartan. A repeat TTE one month later showed LVEF recovery to 55-60% with normal LV wall motion. A repeat troponin was noted to be 23 ng/L.

Given the need for continued chemotherapy, a multidisciplinary decision was made to continue FOLFOX therapy in a monitored setting. The patient was admitted to a telemetry unit and administered a second round of FOLFOX, identical in dosing to the cycle administered one month earlier. She tolerated this well, with no recurrent cardiac events, signs of heart failure, or ischemic changes. She was discharged with oncology follow-up and plans for continued cardiac surveillance during future chemotherapy.

## Discussion

Given that FOLFOX remains a first-line treatment for colorectal cancer, understanding its cardiotoxic risks is critical, particularly in patients with pre-existing cardiovascular disease. While the precise mechanisms by which FOLFOX affects myocardial tissue remain incompletely elucidated, its association with angina, elevated cardiac biomarkers, and electrocardiographic abnormalities concerning myocardial infarction are well documented. Additionally, its potential role in precipitating TCM remains an area of ongoing investigation, signaling the necessity for heightened cardiac surveillance and further research into the biochemical and pathophysiological pathways underlying its cardiotoxicity.

The cardiotoxic effects of 5-FU are believed to result from its conversion into nucleotide metabolites, which interfere with nucleotide and ribonucleotide incorporation into DNA and RNA, respectively [5]. These metabolites disrupt mitochondrial phosphate metabolism, alter calcium channel-dependent membrane function, impair contractile proteins, and generate oxidative stress molecules, all of which contribute to myocardial dysfunction [5]. A primary mechanism underlying 5-FU-induced cardiotoxicity is direct cytotoxic injury to the coronary endothelium, leading to vasoconstriction and coronary spasm. This is supported by in vitro studies demonstrating 5-FU-induced vascular smooth muscle contraction and angiographic findings from case reports that have documented coronary vasospasm following 5-FU administration [2].

Various strategies have been investigated to mitigate the cardiotoxic effects of 5-FU. Studies have shown that protein kinase C (PKC) inhibition may play a role in modulating 5-FU-induced coronary vasospasm. Specifically, the PKC inhibitor staurosporine has been found to attenuate 5-FU-induced vasoconstriction, whereas the PKC agonist phorbol-12,13-dibutyrate exacerbates it, suggesting PKC as a potential therapeutic target [5].

Additionally, plant-derived compounds such as thymoquinone and hesperidin have demonstrated cardioprotective effects against 5-FU-induced oxidative stress by reducing cardiac enzyme levels and minimizing inflammation in preclinical murine models. Notably, the combined use of these agents exhibited superior cardioprotection to either agent alone [6]. Furthermore, *Ganoderma lucidum*, a medicinal mushroom, has shown cardioprotective properties in murine models through an alternative antioxidant mechanism by downregulating heart-type fatty acid-binding protein, malondialdehyde, and cyclooxygenase-2, while upregulating total antioxidant capacity [7]. These findings collectively suggest that oxidative stress modulation is a promising avenue for reducing 5-FU cardiotoxicity. However, limitations remain regarding the long-term effects of these cardioprotective agents in humans and the determination of appropriate dosing regimens for clinical use. Moreover, some studies have alluded to utilizing verapamil or nitrates as prophylactic agents in minimizing the risk of coronary vasospasm in humans, though data supporting their routine use remains limited [2,5].

TCM presents a distinct challenge in cardiology, particularly in patients undergoing chemotherapy, where differentiating stress-induced myocardial dysfunction from direct cardiotoxicity remains complex. While the exact relationship between FOLFOX, 5-FU, and TCM is not fully defined and requires further investigation, evidence suggests that both sympathetic overactivation and chemotherapy-induced oxidative stress may contribute to its pathogenesis. The overlap between 5-FU-induced coronary vasospasm and the microvascular dysfunction seen in TCM raises important questions about their shared mechanisms and potential therapeutic targets. Given the increasing recognition of chemotherapy-related cardiac complications, further research is needed to better characterize the cardiovascular impact of 5-FU and optimize risk mitigation strategies. The potential cardioprotective effects of antioxidant and anti-inflammatory agents, such as thymoquinone, hesperidin, and *Ganoderma lucidum*, exemplify promising avenues for intervention, though clinical validation remains necessary. Moving forward, a cardiology-focused approach incorporating early identification of at-risk patients, preemptive cardioprotective measures, and continuous cardiac monitoring will be essential to improving outcomes in patients receiving FOLFOX chemotherapy.

## Conclusions

As FOLFOX remains a cornerstone of colorectal cancer treatment, its potential for cardiotoxicity necessitates increased awareness, particularly in patients with pre-existing cardiac disease. This case reveals the complex interplay between chemotherapy, coronary vasospasm, and TCM, emphasizing the need for vigilant monitoring and risk mitigation. Baseline troponin values and a transthoracic echo should be obtained prior to FOLFOX initiation. Telemetry monitoring may aid in the early detection of cardiac complications, enabling timely intervention and safer chemotherapy administration. While emerging cardioprotective strategies show promise, further research is essential to refine prevention and management approaches. A proactive, cardiology-driven approach will be key to balancing the efficacy of chemotherapy with the imperative to safeguard cardiac health.
